# Explorative research on glucolipid metabolism and levels of adipokines in pseudohypoparathyroidism type 1 patients

**DOI:** 10.1186/s13023-023-02979-w

**Published:** 2023-11-28

**Authors:** Yi Yang, An Song, Fengying Gong, Yan Jiang, Mei Li, Weibo Xia, Xiaoping Xing, Ou Wang, Hui Pan

**Affiliations:** Department of Endocrinology, Key Laboratory of Endocrinology, Dongcheng District, Peking Union Medical College Hospital, Chinese Academy of Medical Science & Peking Union Medical College, No.1 Shuaifuyuan, Beijing, 100730 China

**Keywords:** Pseudohypoparathyroidism, Glucose metabolism, Lipid metabolism, Adipokine

## Abstract

**Background:**

Pseudohypoparathyroidism type 1 (PHP1) is a rare disease featuring hypocalcemia and elevated PTH level. Though disturbed calcium and phosphorus metabolism under PTH resistant have been widely studied, glucolipid metabolism abnormalities observed in PHP1 patients have received little attention. The aim of this research is to explore the glucolipid metabolism features in a rather large cohort of PHP1 patient. In the current study, PHP1 patients and primary hyperparathyroidism patients as well as normal control were recruited for the investigation. Glucolipid metabolic indices as well as the level of four adipokines were examined.

**Results:**

A total of 49 PHP1 patients, 64 PHPT patients and 30 healthy volunteers were enrolled. A trend of higher HOMA-β index was found in PHP1 patients than normal controls (median 97.08% vs 68.19%, *p* = 0.060). Both the PHP1 and PHPT group presented with significantly lower TNFα level compared to normal controls (average 10.74 pg/ml and 12.53 pg/ml vs 15.47 pg/ml, *p* = 0.002 and 0.041, respectively). FGF21 level was significantly higher in PHPT group than in PHP1 group (median 255.74 pg/ml vs 167.46 pg/ml, *p* = 0.019). No significant difference in glucolipid metabolic indices and adipokines was found between PHP1A or PHP1B patients and normal controls, while overweight/obese PHP1 patients tended to have higher leptin than normal-BMI cases (*p* = 0.055). Multiple linear regression analysis showed BMI rather than PTH or HOMA-IR to be an independent variable of leptin in PHP1.

**Conclusion:**

Metabolic stress given upon especially overweight PHP1 patients may resulted in possible β-cell compensation. Elevated TNFα may be related with hyper-PTH level regardless of calcium level.

## Background

Pseudohypoparathyroidism type 1 (PHP1) is a disease caused by parathyroid hormone (PTH) resistance featuring hypocalcemia, elevated PTH level, unelevated urinary cyclic adenosine monophosphate (cAMP) after exogenous PTH injection, with/without Albright's hereditary osteodystrophy (AHO). Due to loss-of-function mutations of *GNAS* or methylation alteration in differentially methylated regions (DMRs), defected or loss of expression of α-subunit of heterotrimeric guanine-binding protein (Gsα) will reduce the response to PTH at proximal renal tubule, a *GNAS* imprinted expression tissue, defecting calcium reabsorption and 1,25-dihydroxy vitamin D synthesis, thus causing hypocalcemia and elevated PTH level as negative feedback [1].

Calcium-phosphorus metabolic disorders have long been the research focus of PHP1, though its feature on glucolipid metabolism have scarcely been described [2–6]. A relatively higher prevalence of obesity/overweight have been described in one of our former studies [7], and there are also other research emphasizing body fat accumulation in PHP1 patients happening early in life and last throughout lifetime [8].

It is generally believed that adipokines were associated with metabolic status, and some of them could also play a role in glucolipid metabolism regulation. Due to the rarity of PHP1, there still lack evidence on changes of adipokines and its correlation with obesity and abnormal glucolipid metabolism. In this study, we aim to conduct an analysis in a relatively large population of PHP1 patients subtyped with clear molecular subtyping on the associations on levels of adipokines and incidence of overweight/obese as well as glucolipid metabolism indices.

## Results

### Clinical characteristics and basic biochemical features of all subjects

A total of among the 49 PHP1 patients (26 males, 23 females; 25.1 ± 10.8 years) as well as 64 primary hyperparathyroidism (PHPT) patients (13 males, 51 females; 55.5 ± 14.3 years) and 30 normal controls (14 males, 16 females; 26.3 ± 11.2 years) were included in this study (Table 1). The age of the patients in the PHPT group was significantly higher than that of the other two groups, and the female/male ratio was significantly higher than that in the PHP1 group and the normal control group. All the PHP1 patients were under medication of vitamin D and calcium at time of recruitment.

**Table 1:** Demographic characteristics and basic biochemical features of PHP1, normal control and PHPT group

	PHP1 (n = 49)	Normal control (n = 30)	PHPT (n = 64)	p1	p2	p3
M/F (n/n)	26/23	14/16	13/51	0.818	0.014	0.001
Age (years)	25.1 ± 10.8	26.3 ± 11.2	55.5 ± 14.3	0.658	< 0.001	< 0.001
< 18 yo [n, (%)]	11 (22.4%)	9 (30%)	0 (0%)	1.000	0.004	0.001
≥ 18 yo [n, (%)]	38 (77.5%)	21 (70%)	64 (100%)			
BMI (kg/m^2^)	23.88 ± 5.23	21.72 ± 3.07	23.88 (19.47–32.24)	0.117	0.004	0.308
Overweight [n, (%)]	11 (22.4%)	6 (20%)	25 (29.1%)	0.130	–	–
Obese [n, (%)]	7 (14.3%)	0 (0%)	16 (18.6%)			
Overweight/obese [n, (%)]	18 (36.7%)	6 (20%)	41 (64.1%)	0.207	< 0.001	0.004
Ca (mmol/L)	2.24 (1.43–2.50)	2.42 ± 0.07	2.78 ± 0.18	0.048	< 0.001	< 0.001
P (mmol/L)	1.53 ± 0.37	1.19 ± 0.14	0.87 (0.45–1.20)	0.002	< 0.001	< 0.001
ALP (U/L)	76.0 (37.0–443.0)	58.2 ± 15.3	102.0 (54.0–673.0)	< 0.001	< 0.001	< 0.001
24hUCa (mmol)	1.69 (0.10–7.90,n = 42)	NA	7.48 ± 3.13	–	–	< 0.001
β-CTX (ng/ml)	0.34 (0.08–2.90)	0.12 (0.07–0.34)	0.74 (0.05–3.22)	< 0.001	< 0.001	0.108
25OHD (ng/ml)	30.95 (10.30–136.90)	19.16 ± 10.27	17.69 ± 8.98	0.003	1.000	< 0.001
TP1NP (ng/ml)	54.0 (23.9–1141.0)	56.7 (23.8–99.3)	67.7 (13.1–651.1)	0.420	0.126	0.553
PTH (pg/ml)	138.20(10.0–630.3)	37.78 ± 9.58	141.85 (55.7–756.7)	< 0.001	< 0.001	0.181
Cr (μmol/L)	67.69 ± 17.63	74.30 ± 17.00	61.5 (40–134)	0.531	0.024	0.129
Alb (g/L)	47(31–53)	46.00 ± 2.27	45.23 ± 2.73	0.325	0.184	0.022
ALT (U/L)	16 (7–68)	20.83 ± 9.61	17 (7–102)	0.141	1.000	0.022

PHP1: pseudohypoparathyroidism type 1; PHPT: primary hyperparathyroidism; M: male; F: female; BMI: body mass index; Ca: serum calcium; P: phosphorus; ALP: alkaline phosphatase; 24hUCa: 24-h urinary calcium; β-CTX: β-C-terminal telopeptide; 25OHD: 25-hydroxy vitamin D; TP1NP: total procollagen type 1 N-peptide; PTH: parathyroid hormone; Cr: creatinine; Alb: albumin; ALT: alanine aminotransferase; p1: *p*-value for comparison between PHP1 and normal control; p2: *p*-value for comparison between PHPT and normal control; p3: *p*-value for comparison between PHP1 and PHPT. *p*-value of less than 0.05 would be considered as of significantly difference

The body mass index (BMI) of the PHP1 group wasn’t significantly different from normal control and PHPT group (Table 1, *p* = 0.117 and 0.308 respectively), but the BMI of PHPT was significantly higher than normal control (*p* = 0.004). Categorized BMI showed that in PHP1 group, 11 cases (22.4%) were overweight, 7 cases (14.3%) were obese, and the overall ratio of obesity/overweight was not significantly higher than that of the normal control group (*p* = 0.207). The above proportion of PHPT group was significantly higher than that of PHP1 and normal control group (*p* = 0.004 and < 0.001, respectively).

### Indices of glucose and lipid metabolism indices of all subjects

Fasting blood glucose (FBG), insulin (Ins) and homeostatic model assessment for insulin resistance (HOMA-IR) of the PHPT group were significantly higher than those in the normal control group, while homeostatic model assessment for insulin sensitivity (HOMA-IS) was significantly lower than that in the normal control (Table 2; *p* all < 0.05). Medium homeostatic model assessment for β-cell function (HOMA-β) index in the PHP1 group was 97.08%, which was significantly higher than that in the PHPT group (medium level 72.40%, *p* = 0.028), but the difference between the PHP1 group and normal control was not significant (*p* = 0.060).

**Table 2:** Glucolipid metabolic indices of PHP1, normal control and PHPT

	PHP1 (n = 49)	Normal control (n = 30)	PHPT (n = 64)	p1	p2	p3
FBG (mmol/L)	4.80 ± 0.39	4.90 (4.30–6.00)	5.40 (3.80–14.30)	1.000	< 0.001	< 0.001
Ins (μIU/mL)	6.00 (1.90–17.50)	5.57 ± 2.85	7.80 (2.90–19.50)	1.000	0.005	0.031
HOMA-IR	1.24 (0.38–4.04)	1.24 ± 0.66	1.77 (0.71–8.01)	1.000	0.001	0.001
HOMA-IS	0.81 (0.25–2.63)	0.92 (0.35–3.91)	0.57 (0.12–1.41)	1.000	0.001	0.001
HOMA-β (%)	97.08 (26.25–312.50)	80.48 ± 41.53	72.40 (23.33–313.33)	0.060	0.961	0.028
TC (mmol/L)	4.37 ± 0.79	4.18 ± 0.71	4.88 ± 1.05	1.000	0.006	0.012
TG (mmol/L)	0.95 (0.33–2.63)	0.99 (0.31–2.95)	1.17 (0.31–4.22)	0.509	0.126	0.022
Hypertriglyceridemia [n, (%)]	8 (16.3%)	3 (10%)	13 (20.3%)	0.523	0.255	0.631
HDL-C (mmol/L)	1.26 (0.77–2.19)	1.26 (0.85–2.27)	1.32 ± 0.31	0.777	0.930	0.946
Hypo-HD-lipoproteinemia [n, (%)]	3 (6.1%)	1 (3.3%)	5 (7.8%)	0.146	0.707	0.228
LDL-C (mmol/L)	2.61 ± 0.62	2.62 ± 0.58	2.86 ± 0.94	0.616	0.150	0.045
Hyper-LD-lipoproteinemia [n, (%)]	5 (10.2%)	3 (10%)	16 (25%)	0.509	0.309	0.052
Leptin (ng/ml)	2.43 (0.10–6.39)	1.16 (0.09–9.02)	2.24 (0.10–8.24)	1.000	0.037	0.124
Adiponectin (μg/ml)	4.64 (2.27–81.80)	5.39 ± 3.05	4.95 (1.80–12.24)	0.889	0.964	0.977
FGF21 (pg/ml)	167.46 (17.82–975.23)	165.29 (63.19–649.68)	255.74 (45.59–847.88)	1.000	0.094	0.019
TNFα (pg/ml)	10.74 ± 4.13	15.47 ± 6.65	11.48 (3.82–44.61)	0.002	0.041	0.481
LAR	0.19 (0.01–3.19)	0.34 (0.02–2.26)	0.50 (0.01–2.24)	0.604	0.062	0.141

PHP1: pseudohypoparathyroidism type 1; PHPT: primary hyperparathyroidism; FBG: fasting blood glucose; Ins: insulin; HOMA-IR: homeostasis model assessment-insulin resistance; HOMA-IS: homeostasis model assessment-insulin sensitivity; HOMA-β: homeostasis model assessment-beta cell function; TC: total cholesterol; TG: triglyceride; HDL-C: high density lipoprotein cholesterol; LDL-C: low density lipoprotein cholesterol; FGF21: fibroblast growth factor 21; TNFα: tumor necrosis factor α; LAR: leptin/adiponectin ratio; p1: *p*-value for comparison between PHP1 and normal control; p2: *p*-value for comparison between PHPT and normal control; p3: *p*-value for comparison between PHP1 and PHPT. *p*-value of less than 0.05 would be considered as of significantly difference

There was no significant difference in total cholesterol (TC), triglyceride (TG), high density lipoprotein cholesterol (HDL-C) and low-density lipoprotein cholesterol (LDL-C) between the PHP1 group and the normal control group (p all > 0.05), but the TC and LDL-C levels in the PHPT group were significantly higher than those in the PHP1 (4.88 ± 1.05 mmol/L vs 4.37 ± 0.79 mmol/L and 2.86 ± 0.94 mmol/L vs 2.61 ± 0.62 mmol/L, *p* = 0.012 and 0.045 respectively). However, there was no significant difference in the proportion of hypertriglyceridemia, elevated LDL-C level or lowered HDL-C level among the three groups.

### Serum adipokines levels of all subjects

The serum tumor necrosis factor α (TNFα) level of PHP1 group was significantly lower than normal control group (average 10.74 pg/ml vs 15.47 pg/ml, *p* = 0.002), while other serum adipokine levels didn’t show significant difference. Fibroblast growth factor 21 (FGF21) level was significantly lower in PHP1 than in PHPT (median 167.46 pg/ml vs 255.74 pg/ml, *p* = 0.019). Although TNFα level was significantly lower in PHPT than normal control (medium 11.48 pg/ml vs 14.72 pg/ml, *p* = 0.041), leptin of PHPT was significantly higher (medium 2.24 pg/ml vs 1.16 pg/ml, *p* = 0.037). As for calculated leptin/adiponectin ratio (LAR), the medium LAR of PHP1, normal control and PHPT group were 0.19, 0.34 and 0.50, where no significant difference between groups was found (p all > 0.050).

### Comparison of glucolipid metabolism indexes and adipokines levels among different PHP1 subgroups

There was no difference in age and gender distribution between PHP1A and PHP1B subgroups (Table 3, *p* all > 0.050), but the BMI of the PHP1A group tended to be higher than that of the normal control group (*p* = 0.050), though there was no significant difference in the proportion of overweight/obese people between PHP1A, PHP1B and the normal control group. There were 5 underage patients in the PHP1A group who were overweight/obese comparing with children of the same gender and age. Among the 9 adult PHP1A patients, 5 were overweight/obese, accounting for 55.6% of them.

**Table 3:** Demographic and glucolipid metabolic indices of PHP1 patients of different molecular subgroups

	PHP1 (n = 49)	PHP1A (n = 14)	PHP1B (n = 35)	Normal control (n = 30)	p1	p2	p3
M/F (n/n)	26/23	8/6	17/18	14/16	0.758	1.000	0.758
Age (years)	25.1 ± 10.8	25.1 ± 8.0	27.4 ± 10.2	25.1 ± 10.8	0.682	0.524	1.000
< 18 yo [n, (%)]	11 (22.4%)	5 (35.7%)	6 (17.1%)	9 (30%)	1.000	0.251	0.269
≥ 18 yo [n, (%)]	38 (77.5%)	9 (64.3%)	29 (82.9%)	21 (70%)			
BMI (kg/m^2^)	23.88 ± 5.23	25.14 ± 5.72	23.20 ± 4.63	21.72 ± 3.07	0.050	0.143	0.332
Overweight [n, (%)]	11 (22.4%)	3 (21.4%)	8 (22.9%)	6 (20%)	-	-	0.627
Obese [n, (%)]	7 (14.3%)	3 (21.4%)	4 (11.4%)	0 (0%)			
Overweight/obese [n, (%)]	18 (36.7%)	6 (42.8%)	12 (34.3%)	6 (20%)	0.174	0.269	0.754
FBG (mmol/l)	4.80 ± 0.39	4.61 ± 0.32	4.84 ± 0.38	4.90 (4.30–6.00)	0.943	1.000	1.000
Ins (μIU/ml)	6.00 (1.90–17.50)	5.40 (1.90–13.80)	6.70 (2.10–17.50)	5.57 ± 2.85	1.000	1.000	1.000
TG (mmol/L)	0.95 (0.33–2.63)	1.16 ± 0.74	1.04 ± 0.48	0.99 (0.31–2.95)	0.866	0.434	0.751
LDL-C (mmol/L)	2.61 ± 0.62	2.25 (1.45–4.04)	2.64 ± 0.55	2.62 ± 0.58	0.413	0.828	0.363
HOMA-IR	1.24 (0.38–4.04)	1.34 ± 0.79	1.35 (0.48–4.04)	1.24 ± 0.66	1.000	1.000	1.000
HOMA-IS	0.81 (0.25–2.63)	0.83 (0.37–2.63)	0.75 (0.25–2.10)	0.92 (0.35–3.91)	1.000	1.000	1.000
HOMA-β (%)	97.08 (26.25–312.50)	108.00 (38.00–306.67)	94.79 (26.25–312.50)	80.48 ± 41.53	0.155	0.135	0.680
Leptin (ng/ml)	2.43 (0.10–6.39)	1.57 (0.12–6.17)	2.48 (0.10–6.39)	1.16 (0.09–9.02)	0.753	0.398	0.849
Adiponectin (μg/ml)	4.64 (2.27–81.80)	5.22 ± 2.19	4.57 (2.58–81.80)	5.39 ± 3.05	0.828	0.948	0.941
FGF21 (pg/ml)	167.46 (17.82–975.23)	163.69 (59.31–975.05)	154.45 (17.82–693.97)	165.29 (63.19–649.68)	0.904	0.596	0.528
TNFα (pg/ml)	10.74 ± 4.13	10.51 ± 4.05	10.82 ± 4.29	15.47 ± 6.65	0.013	0.002	0.801
LAR	0.34 (0.02–2.26)	0.65 ± 0.78	0.38 (0.02–1.81)	0.19 (0.01–3.19)	0.648	0.673	1.000

PHP1: pseudohypoparathyroidism type 1; PHP1A: PHP1: pseudohypoparathyroidism type 1A; PHP1: pseudohypoparathyroidism type 1B; PHPT: primary hyperparathyroidism; M: male; F: female; BMI: body mass index; FBG: fasting blood glucose; Ins: insulin; TG: triglyceride; LDL-C: low density lipoprotein cholesterol; HOMA-IR: homeostasis model assessment-insulin resistance; HOMA-IS: homeostasis model assessment-insulin sensitivity; HOMA-β: homeostasis model assessment-beta cell function; FGF21: fibroblast growth factor 21; TNFα: tumor necrosis factor α; LAR: leptin/adiponectin ratio; p1: *p*-value for comparison between PHP1A and normal control; p2: *p*-value for comparison between PHP1B and normal control; p3: *p*-value for comparison between PHP1A and PHP1B. *p*-value of less than 0.05 would be considered as of significantly difference

There was no significant difference in the glucolipid metabolism indices between different molecular subtype subgroups of PHP1 and the normal control group. TNFα level comparisons between PHP1A and PHP1B subgroups with normal control group was consistent with the results observed in total PHP1 group, while the TNFα levels between the two subgroups were similar (Table 3, *p* = 0.801). There was no significant difference in the levels of serum leptin, adiponectin and FGF21 as well as the calculated LAR between the two subgroups or normal control group.

The overweight/obese subgroup presented with significantly higher levels of Ins, HOMA-IR, HOMA-β, and significantly lower HOMA-IS comparing with normal BMI subgroup (Table 4, *p* = 0.033, 0.023, 0.005 and 0.023, respectively). But the lipometabolic indices of the overweight/obese subgroup weren’t of significant difference from the normal control group or the normal BMI subgroup.

**Table 4:** Glucolipid metabolic indices of PHP1 patients of different BMI subgroup

	PHP1-normal BMI (n = 31)	PHP1-overweight/obese (n = 18)	Normal control (n = 30)	p1	p2	p3
FBG (mmol/l)	4.69 ± 0.31	4.78 ± 0.46	4.90 (4.30–6.00)	0.336	0.404	0.951
Ins (μIU/ml)	4.85 (2.20–17.50)	8.12 ± 3.79	5.57 ± 2.85	0.833	0.028	0.033
TG (mmol/L)	0.92 (0.39–2.12)	1.17 ± 0.59	0.99 (0.31–2.95)	0.433	0.798	0.724
LDL-C (mmol/L)	2.51 ± 0.42	2.80 ± 0.71	2.62 ± 0.58	0.300	0.624	0.237
HOMA-IR	1.08 (0.50–4.04)	1.85 ± 0.95	1.24 ± 0.66	0.597	0.032	0.023
HOMA-IS	1.00 ± 0.48	0.64 (0.29–1.80)	0.92 (0.35–3.91)	0.592	0.032	0.023
HOMA-β (%)	98.45 (42.5–312.50)	121.33 (60–306.67)	80.48 ± 41.53	0.683	0.002	0.005
Leptin (ng/ml)	0.29 (0.11–4.79)	3.11 ± 2.00	1.16 (0.09–9.02)	0.785	0.055	0.029
Adiponectin (ng/ml)	5.96 (2.58–14.23)	4.30 (2.27–81.80)	5.39 ± 3.05	0.612	0.639	0.196
FGF21 (pg/ml)	162.84 (17.82–693.97)	133.44 (25.70–975.05)	165.29 (63.19–649.68)	0.805	0.338	0.272
TNFα (pg/ml)	12.18 ± 5.01	10.31 ± 3.78	15.47 ± 6.65	0.004	0.005	0.914
LAR	0.05 (0.02–1.49)	0.77 ± 0.67	0.19 (0.01–3.19)	0.297	0.115	0.029

PHP1: pseudohypoparathyroidism type 1; BMI: body mass index; FBG: fasting blood glucose; Ins: insulin; TG: triglyceride; LDL-C: low density lipoprotein cholesterol; HOMA-IR: homeostasis model assessment-insulin resistance; HOMA-IS: homeostasis model assessment-insulin sensitivity; HOMA-β: homeostasis model assessment-beta cell function; FGF21: fibroblast growth factor 21; TNFα: tumor necrosis factor α; LAR: leptin/adiponectin ratio; p1: *p*-value for comparison between PHP1-normal BMI and normal control; p2: *p*-value for comparison between PHP1-overweight/obese and normal control; p3: *p*-value for comparison between PHP1-normal BMI and PHP1- overweight/obese. *p*-value of less than 0.05 would be considered as of significantly difference

The level of serum leptin of the overweight/obese subgroup was significantly higher than that of the normal BMI subgroup (medium 3.08 ng/ml vs 0.29 ng/ml, *p* = 0.029), and it tended to be higher than that of the normal control group (*p* = 0.055). The serum TNFα levels of both overweight/obese subgroup and normal BMI subgroup were significantly lower than the normal control group (*p* = 0.004 and 0.005 respectively), which was consistent with the difference between the overall serum TNFα level of PHP1 patients and the normal control group. The calculated LAR for normal BMI subgroup was significantly higher than that of the overweight/obese subgroup (medium 0.34 vs 0.19, *p* = 0.029), when there were no significant differences between different BMI subgroups and normal control group.

**Fig. 1:**
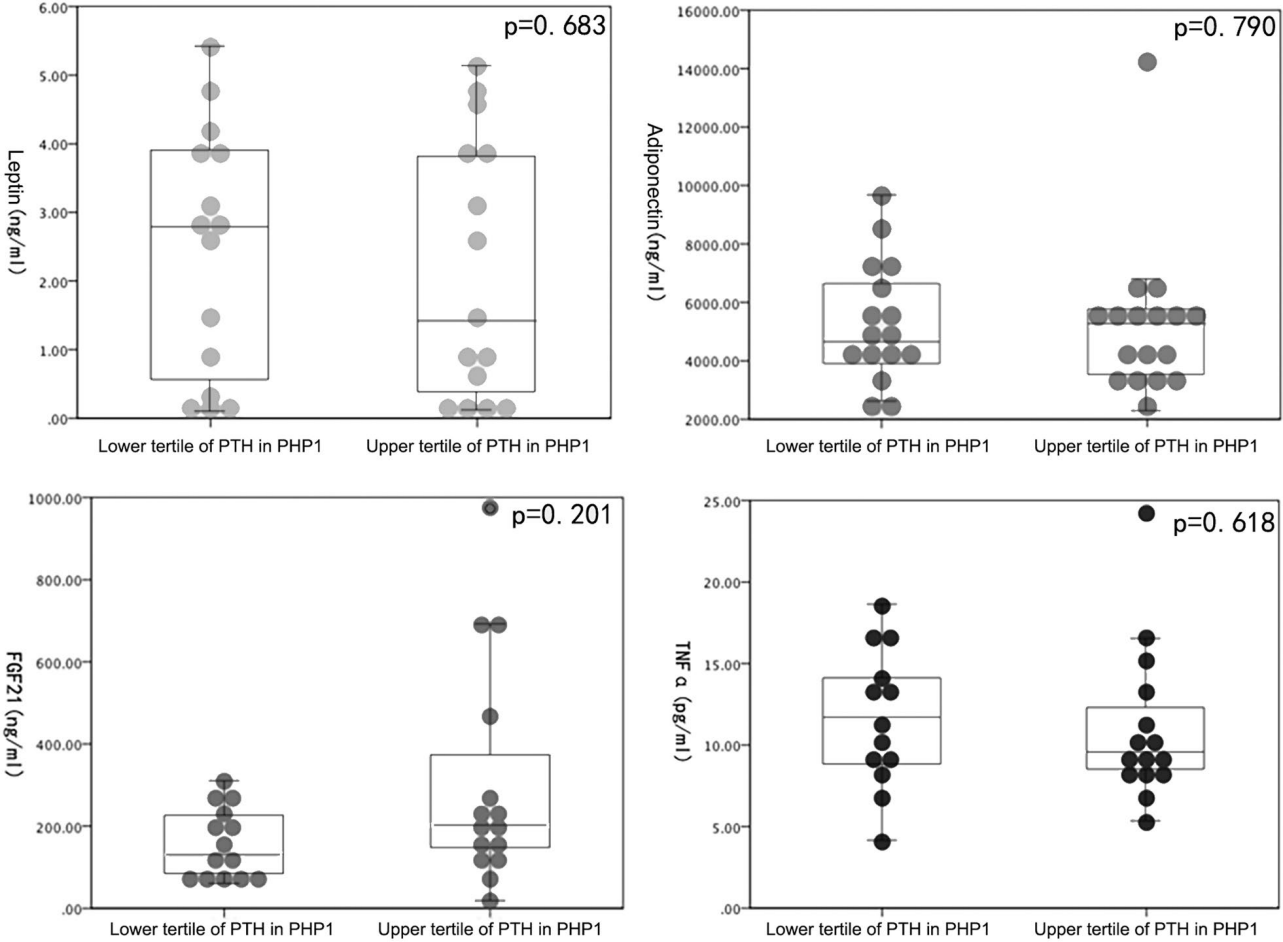
Scatter plot and box plot of adipokine levels in the lower and upper tertile of PTH in PHP1 patients. The patients of PHP1 were divided according to the tertiles of PTH levels, and adipokine levels were compared and plotted between the subgroups of patients with the PTH level of the lower and upper tertile. Lower tertile of PTH in PHP1: PHP1 patients with PTH level of 10.0–89.3 pg/ml. Upper tertile of PTH in PHP1: PHP1 patients with PTH level of 193.3–630.3 pg/ml. PHP1: pseudohypoparathyroidism; PTH: parathyroid hormone; FGF21: fibroblast growth factor 21; TNFα: tumor necrosis factor α. *P*-value of less than 0.05 would be considered as of significantly difference

We further divided PHP1 patients into three subgroups according to the tertiles of PTH level: subgroup of lower tertile of PTH (ranged 10.0–89.3 pg/ml), subgroup of medium tertile of PTH (ranged 92.0–157.7), and subgroup of upper tertile of PTH (ranged 193.3–630.3 pg/ml). Then the levels of the adipokines of the lower tertile of PTH subgroup were compared against those of the upper tertile of PTH subgroup. The results of the comparison were further plotted in Fig. 1. There were no significant differences in serum leptin, adiponectin, FGF21 or TNFα levels between the upper tertile subgroup and the lower tertile subgroup (Fig. 1, *p* = 0.683, 0.790, 0.201 and 0.618 respectively).

### Comparison of glucolipid metabolism indexes and adipokines levels among different PHP1 subgroups

Multiple linear regression was applied for further analyze of independent influencing factor of serum leptin levels. BMI, PTH and HOMA-IR were selected as independent variables. Multiple linear regression showed that with BMI, PTH, and HOMA-IR as independent variables, the model was statistically significant (F = 6.452, *p* = 0.002, adjusted R^2^ = 0.614). Among the three independent variables, the effect of BMI on the leptin level of the PHP1 group was statistically significant (*p* = 0.001), therefore BMI could be considered as independent variable of leptin of PHP1 group. Further inclusion of serum Ca and 25OHD as another two independent variables have no influence on the impact of the above three variables on leptin level.

## Discussion

This study provided a broader view of glucolipid metabolism in PHP1 based on a rather large cohort of Chinese PHP1 patients. By evaluating the metabolic indices and adipokine levels, we found that PHP1 patients who were overweight/obese presented with abnormal HOMA indices as well as TNFα and leptin levels, which have not been revealed in previous research concerning similar clinical issues.

In our study, the overall overweight/obesity ratio in PHP1 group and PHP1A, PHP1B subgroups were 36.7%, 42.8% and 34.3%, respectively, which were not significantly different from the normal control group. One of our previous researches found that the overweight/obese rate were 40% and 38% for 10 PHP1A/1C and 94 PHP1B patients, respectively, which were higher than the general population [7]. One recent large cohort survey recruited 2548 underage patients with severe obesity and conducted genetic analysis. Among them, 22 presented with early-onset obesity, and among whom 19 bore heterozygous *GNAS* mutation, revealing an unexpectedly high prevalence of loss-of-function *GNAS* mutation among junior obesity patients [11]. The small sample size may be responsible for the disparity between current study and previous research, which means that although the prevalence may seem similar, as one of the characters of AHO, overweight/obese can still be part of the concerning health problems for PHP1 patients.

Previous studies implicated the occurrence of higher incidence of underage obesity [5] and early-onset obesity as early as the first year of life [8] among PHP1 especially PHP1A patients, which was not observed in our study since the number of patients with/without overweight/obese was not significantly different in different age subgroups. One reason may be that underage patients, especially infants with early-onset PHP1 related symptoms were referred to specialized hospitals, leading to a selection bias in our study. The bias also resulted in a relatively elder age distribution in the underage group, whose diet and eating behavior had been greatly affected by education and dietary regulation rather than their underlying genetic defects, which may be another possible reason for the unobvious overweight/obese prevalence in the underage PHP1 patients.

To date, there are only a few case reports describing hyperinsulinemia and impaired glucose tolerance in PHP1 patients [2–4]. Two recent cohort studies evaluated the glucometabolism in adult and underage PHP1A patients, revealing glucose tolerance impairment, β-cell dysfunction and delayed appearance of blood glucose peaks, slower rate of postprandial blood glucose decline in these two groups respectively [5, 6], all of which indicated deeper investigation into the reason and risk factors of the underlying abnormal glucometabolism in PHP1 patients.

While pancreatic β-cell function was described to decrease in PHP1A patients in previous research [5, 6], the higher HOMA-β observed in the in overweight/obese subgroup of PHP1 could be seen as a compensational increase in β-cell function under glucolipid metabolic stress during the early stage of type 2 diabetes according to what Bonner-Weir et al. had described [12, 13], which were later proved by Puff et al. [14]. Although previous studies have confirmed that the effects of some G protein coupled receptor (GPCR) medicating incretin lost their insulin stimulating function in Gsα knockout mice [15], the present study showed no difference of glucometabolism indexes between different molecular subtypes of PHP1 patients. The abnormality in overweight/obese but not in certain molecular subtype of PHP1 patient suggested that the impact of body fat accumulation exceeded the deviant expressed *GNAS* in insulin stimulation, inducing the compensation of β-cell function in these patients. However, only fasting blood glucose and fasting insulin were evaluated in our study. For further investigation, OGTT, IVGTT and glucose-insulin clamp test could act as a more effective and sensitive method for glucometabolism evaluation in PHP1 patients in the future.

In our study, PHP1 patients presented with unparticular serum lipid levels comparing with normal control group, though TC, LDL-C, and HDL-C levels were significantly lower than those in the PHPT group. The correlation of high PTH concentration and hyperlipidemia have only been observed in PHPT population [16], while in PHP1, the defected GPCR pathway may be a concerning factor for the non-responding GPCR-dependent lipolysis pathway of PTH [16]. The medication of calcitriol in PHP1 patients could also influence the lipolysis of lipoprotein lipase (LPL) [17], which eventually resulted in a seemingly normal level of serum lipid level in PHP1 patients.

Analysis on leptin levels revealed the relationship between BMI and leptin which was observed to be significantly higher in overweight/obese subgroup of PHP1 patients. Apart from its correlations with obesity [18], it’s also believed that leptin could serve as a regulator of satiety mediated by GPCR in hypothalamus [19]. But the difference in LAR rest between BMI-based subgroups but not molecular subgroups suggested that the impact of body fat accumulation on peripheral leptin level may out-weighted the negative feedback regulation of the leptin-melanocortin pathway due to the malfunction of Gsα. The BMI related differences in LAR further suggested the possible insulin resistance, as previous investigation on elder female and severely obese population have associated higher LAR with worse insulin sensitivity [20, 21], and the similarity in LAR between PHP1A and PHP1B group could be explained by the indifferent BMI of them.

The PHPT group in our research preserved a significantly higher level of leptin than the other two groups. First of all, the elder age in the PHPT patient group may serve as an unneglectable reason for their higher BMI and the alteration in glucolipid metabolic features. Moreover, Maetani et al. found that within a rather large sample study, the relatively high blood 25OHD level had a correlation with low leptin level [22], suggesting that the difference in 25OHD in this study may be responsible for the difference in leptin between PHP1 and PHPT group. One recent in vitro study found that the leptin expression to be elevated in parathyroidectomized PHPT tissues, and PTH could be further produced when leptin was taken up [23]. Therefore, elevated leptin may as both the result of PTH induced fat accumulation and the stimuli on PTH production, thus contributing to the “PTH elevation-weight gain-leptin expression” cycle, which will aggravate glucolipid metabolism abnormality.

In our study, FGF21 levels were significantly higher in PHPT than in the other two groups. The correlation of age and FGF21 level [24] could explain the relatively higher FGF21 level in PHPT group. Although in PHP1 patients, due to the imprinting defect of β-adrenergic receptor in BAT [25], the cold stimuli on BAT via the β-adrenergic receptor-cAMP pathway [26] was weakened caused by the decreased expression/dysfunction of Gsα, presented as impaired elevation in FGF21 secretion. But the speculation still needs to be verified by further experiments.

Our findings showed significantly lower TNFα levels in the PHP1 and PHPT group than in the normal control group. Previous research found a significantly lower serum TNFα level in the vitamin D supplementary group comparing with the control group within a cohort of type 2 diabetes patients [27], suggesting that the vitamin D supplementation may have contributed to this difference. Furthermore, a newly published research involving meniscus injury induced osteoarthritis (MIO) model mice have observed a significant decrease in TNFα in PTH(1–84) treated MIO mice chondrocyte in vitro [28]. Considering the anti-inflammatory effect of PTH, the elevated serum PTH level could be a plausible reason for a lower peripheral TNFα concentration in both PHP1 and PHPT patients. But it is worth noting that the lowering of only TNFα should not be interpreted as the sign of a lower inflammation grade in PHP1 than in healthy population. Subsequent studies involving more factors are needed in order to provide a complete picture of the abnormal glucolipid metabolism and chronic inflammatory response due to overweight/obesity in PHP1 and PHPT patients.

Some limitations of our study should be mentioned. First of all, due to the rarity of the disease and the selection bias, the cohort of PHP1 was rather small, and the age was relatively elder, making the difference between obese/non-obese as well as underage/adult not as apparent as it was described in previous studies. The selection of the detected adipokines was mostly based on the general knowledge over obesity. Further analysis on more BAT-specified adipokines is needed for a deeper understanding on the influence of *GNAS* molecular defection on the function of adipose tissue.

## Conclusion

Our research suggested the glucometabolic stress given upon overweight/obese PHP1 patients by fat accumulation resulting in β-cell compensation. The impact of obesity was also indicated by the trend of higher level of leptin observed in this subgroup of PHP1 patients compared to normal controls. The higher TNFα level in PHP1 and PHPT groups could be a previously scarcely noticed consequence related with elevated PTH levels, though medication including calcitriol may still had certain impact in especially PHP1 patients. The impact of elder age of the group PHPT should not be neglected, which may also explain the higher BMI and abnormal glucolipid features. PHP1 patients had high prevalence of overweight/obese, while its association with glucolipid metabolisms still needs larger sized study with more comprehensive and sensitive markers. Further studies focusing BAT-specific adipokine and glucolipid metabolic pathway would be needed for a throughout understanding observation of the clinical spectrum of PHP1. More evidence is needed for making a conclusive explanation on whether *GNAS* mutation/methylation alteration may serve as an underlying reason.

## Materials and methods

### Participants

PHP1 patients as well as PHPT patients were recruited at the out-patient division of Endocrinology, Peking Union Medical College Hospital. All clinical investigations and medications were conducted according to the standard clinical procedures at our center. Diagnosis of PHP1 was established upon hypocalcemia, elevated PTH level and typical clinical manifestations such as epilepsy, tetany, with/without signs of AHO, etc. Exclusion criteria were idiopathic or secondary hypoparathyroidism, secondary hyperparathyroidism, abnormal renal function, severe liver failure, and/or had used medications that could affect calcium/phosphorus metabolism except for calcium and vitamin D. PHPT was diagnosed based on hypercalcemia (serum calcium over 2.70 mmol/L or ionized calcium over 1.28 mmol/L) with an uninhibited PTH level. Exclusion criteria for PHPT were secondary hyperparathyroidism, history of parathyroidectomy, severe liver failure, kidney dysfunction, and/or the usage of medications that could affect calcium/phosphorus metabolism except for calcium and vitamin D. The normal control group was consisted of three parts in order to be gender- and age- matching with PHP1 group. Part of the volunteers who were over 18 years old were recruited from healthy adults who visited our hospital for routine physical examination; another part of the adult volunteers’ sample was from the Clinical Biobank, Peking Union Medical College Hospital, Chinese Academy of Medical Sciences. The underage controls were recruited from primary and secondary school students from Pinggu District, Beijing. Volunteers with metabolic bone disease, severe liver or renal failure, severe cardiovascular disease, diabetes mellitus or obesity were excluded. Written informed consent was obtained from the participants or their guardians for genetic analysis. This study was approved and supervised by ethics committee of PUMCH (Ethics Approval registration number JS-3314).

### Clinical information

Detailed clinical information of PHP1 patients was obtained retrospectively through the Hospital Information System of PUMCH, and some unrecorded information was obtained through telephonic follow-up. Basic information including gender, age, weight and height were recorded, and body mass index (BMI) was calculated by body weight (weighted in kilogram) divided by squared height (measured with meter). BMI was further divided into three categories (normal, overweight, obese) according to standard criteria of different age groups of Chinese [9]. Current medical treatment (dose of vitamin D and calcium) was recorded.

### Subtyping of PHP1 patients

Peripheral blood samples were collected additionally among PHP1 patients (Cat.367856, BD Vacutainer® Blood Collection Tubes, BD, US), from which DNA samples were extracted using a commercial kit (Cat.D3494, E.Z.N.A. Blood DNA Midi Kit, Omega Bio-tek, US). Confirmation and subtyping of PHP1 were done with DNA samples by conducting methylation-specific multiplex ligation-dependent probe amplification (MS-MLPA) and *GNAS* analysis [methods described in previous studies [7, 10]]. Patients of PHP1 with clear molecular subtyping would be included in our research, and their laboratory information will be recorded as described above.

### Laboratory examination

Laboratory examination were conducted at the Department of Laboratory, PUMCH. Serum calcium (Ca), phosphorus (P), 24-h urinary calcium (24hUCa), alkaline phosphatase (ALP), alanine aminotransferase (ALT), creatinine (Cr), fasting blood glucose (FBG), total cholesterol (TC), triglyceride (TG), high-density lipoprotein cholesterol (HDL-C) and low-density lipoprotein cholesterol (LDL-C) were measured with automated analyzer (Beckman Coulter AU5800, USA). Serum parathyroid hormone (PTH), 25-hydroxy vitamin D (25OHD), β-C-terminal telopeptide (β-CTX), total procollagen type 1 N-peptide (TP1NP) and fasting insulin (Ins) were measured with electrochemiluminescent immunoassay system (Roche Cobas e601). Homeostasis model assessment-insulin resistance (HOMA-IR), homeostasis model assessment-insulin sensitivity (HOMA-IS) and homeostasis model assessment-beta cell function (HOMA-β) were calculated according to following formulas:

$${\text{HOMA}} - {\text{IR}} = \frac{{{\text{FBG}}\;{\text{mmol}}/{\text{L}} \times {\text{Ins}}\;\upmu {\text{IU}}/{\text{mL}}}}{22.5}$$

$${\text{HOMA}} - {\text{IS}} = \frac{22.5}{{{\text{FBG}}\;{\text{mmol}}/{\text{L}} \times {\text{Ins}}\,\upmu {\text{IU}}/{\text{mL}}}}$$

$${\text{HOMA}} -\upbeta = \frac{{{\text{Ins}}\;\upmu {\text{IU}}/{\text{mL}} \times 20}}{{{\text{FBG}}\;{\text{mmol}}/L - 3.5}} \times 100\%$$

### Determination of adipokine concentration

Commercially available enzyme-linked immunosorbent assay (ELISA) kits were used to measure serum leptin, TNF-α, adiponectin, (Kit No. SEA084Hu, SEA133Hu, and SEA605Hu, USCN Life Science, China) and FGF21 (Kit No. ab222506, Abcam, United Kingdom) concentrations (the intra-assay variance and inter-assay variance were < 10% and < 12% for leptin, TNFα and adiponectin; < 5.3% and < 5.5% for FGF21, respectively). Furthermore, leptin/adiponectin ratio (LAR) were also calculated by leptin level divided by adiponectin level.

### Statistical analysis

All results were analyzed by SPSS24. All data that were normally distributed were described by the mean and standard deviation. Non-normally distributed data were described as the median and minimum and maximum ranges. Non-continuous variables were described by the number of cases (proportion%). Categorical variables were described by the number of cases (proportion%). Student t-test was used to examine the differences of groups of normally distributed data, and non-parametric test was used to examine the differences between groups of non-normally distributed data. Multiple linear regression was applied for further analyze of independent influencing factor of serum leptin levels. BMI, PTH and HOMA-IR were selected as independent variables. A *p*-value lower than 0.05 would be considered statistically significant.

## Data Availability

The datasets used and/or analyzed during the current study are available from the corresponding author on reasonable request.
